# Human mitochondrial genome compression using machine learning techniques

**DOI:** 10.1186/s40246-019-0225-3

**Published:** 2019-10-22

**Authors:** Rongjie Wang, Tianyi Zang, Yadong Wang

**Affiliations:** 1 Peng Cheng Laboratory, ShenZhen, China; 2 School of Computer Science and Technology, Harbin Institute of Technology, Harbin, China

**Keywords:** Compression, Human mitochondrial genomes, Machine learning

## Abstract

**Background:**

In recent years, with the development of high-throughput genome sequencing technologies, a large amount of genome data has been generated, which has caused widespread concern about data storage and transmission costs. However, how to effectively compression genome sequences data remains an unsolved problem.

**Results:**

In this paper, we propose a compression method using machine learning techniques (DeepDNA), for compressing human mitochondrial genome data. The experimental results show the effectiveness of our proposed method compared with other on the human mitochondrial genome data.

**Conclusions:**

The compression method we proposed can be classified as non-reference based method, but the compression effect is comparable to that of reference based methods. Moreover, our method not only have a well compression results in the population genome with large redundancy, but also in the single genome with small redundancy. The codes of DeepDNA are available at https://github.com/rongjiewang/DeepDNA.

## Background

The Human Genome Project (HGP) cost about $3 billion and took about 13 years, the completion of the Human Genome Project signs of the beginning of human genome research in the life sciences has entered a new era of genome [1]. Since then, The amount of data in the genome is growing exponentially, even faster than Moore’s Law [2]. Today’s high-throughput sequencing technology enables sequencing of individual genomes in a matter of hours, with sequencing costs less than $1,000. These advances have allowed researchers to increase the scope for scientific discovery through large amounts of data. However, the huge amount of genomic data presents new challenges for efficient storage and transmission.

Genome sequences are generally stored in FASTA format [3]. In this format, genome sequence characters are stored in ASCII-based, and represented by four diiferent symbols (called nucleotides or bases), namely (A) adenine, (C) cytosine, (T) thymine, (G) guanine. The problem we face is how to effectively compress strings of a certain length composed of these four elements.

The existing genome compression methods were major based on the dictionary methods [4–7], and based on statistical methods [8, 9]. DeepZip [10], as a machine learning compression method, compression the general context data at a online learning model. The parameters of compression is updated once after compressing each character, trying to learn the pattern of input data. The consequence of this approach is that a lot of time is spent updating model parameters during compression and decompression. Another minor defect is that when a pattern first appears in the input data, the compression and decompression model cannot be recognized it immediately. It needed to learn the pattern, and then, can be effectively compressed when these pattern were encountered again, which affects the compression result. As we know that genomes within the same species have a highly similarity, for example, genome similarity between two human individuals up to 99.9*%*. Even not in the same species, the genome similarity between humans and chimpanzees can be as high as 89% [11]. The banana genome, which seems to have nothing to do with the human genome, has a similarity of 50% [12]. In this work, we proposes a static machine learning compression method, use part of experiment data as a training set, optimal the compression model parameters. Then use the other part as test data to test the effect of compression model.

In computer vision tasks, the deep learning model has achieved some good performance, such as using Convolutional Neural Network (CNN) and Long Short-Term Memory Networks (LSTM) models to solve text classification [13], image caption generation [14] and speech recognition [15], and so on. However, in genome sequence data compression, for the first time, we tried to use a deep learning model to learn the sequence of patterns in the genome, and to predict the probabilities of the next base to be encoded, followed by arithmetic coding and output compressed data stream.

We verified the validity of our proposed method in 1,000 human mitochondrial sequences, and randomly divided the data set into three parts in proportion (training set, verification set and test set). Then we trained our model with the training set, the verification set was used to select the optimal parameters, and the test set verified the effectiveness of the deep learning model.

The remainder of this paper is organized as follows: Section II describes the DeepDNA method in detail, section III reports the experimental performance of DeepDNA, conclusion is drawn in Section IV.

## Methods

### Overview

For a length of T sequence steam *x*_1:*T*_=*x*_1_,*x*_2_,…,*x*_*T*_, where each variable *x*_*i*_∈*Σ*,*i*∈[ 1,*T*], for genome sequence, *Σ*={*A*,*C*,*G*,*T*}. The probability of the entire sequence *x*_1:*T*_is:

1 $$ \begin{aligned} p\left({x}_{1 : T}\right) &=p\left(x_{1}\right) p\left(x_{2} |{x}_{1}\right) p\left(x_{3} | {x}_{1 : 2}\right) \cdots p\left(x_{T} | {x}_{1 :(T-1)}\right) \\ &=\prod_{t=1}^{T} p\left(x_{t} | {x}_{1 :(t-1)}\right) \end{aligned}  $$

Therefore, the probability density estimation problem of sequence data can be converted into a univariate conditional probability estimation, that means, the probability of a sequence can be viewed as the product of its probability of sub-sequence. The more likely a sequence is to occur, the lower its entropy value and the better compression result. In other words, the more accurate the prediction of conditional probability events of sub-sequence, the better the compression effect achieved. The conditional probability can be expressed as *p*(*x*_*t*_|*x*_1:(*t*−1)_) of *x*_*t*_ given *x*_1:(*t*−1)_, which fed into the arithmetic encoding tool, to get the final compressed file.

Given N sequences data, the sequence probability model needs to learn a model *p*_*θ*_(*x*_*t*_|*x*_1:(*t*−1)_), to maximize the log likelihood function of the entire data set.

2 $$ \max_{\theta} \sum_{n=1}^{N} \log p_{\theta}\left({x}_{1 : T_{n}}^{(n)}\right)=\max_{\theta} \sum_{n=1}^{N} \sum_{t=1}^{T_{n}} \log p_{\theta}\left(x_{t}^{(n)} | {x}_{1:(t-1)}^{(n)}\right)  $$

We can use the neural network model to estimate the conditional probability *p*_*θ*_(*x*_*t*_|*x*_1:(*t*−1)_). Suppose a neural network *f*(*θ*), whose input is the historical information *x*_1:(*t*−1)_=*x*_1_,*x*_2_,…,*x*_*t*−1_, the output is the occurrence of next base *x*_*t*_, the output four nucleotides probabilities satisfy:

3 $$ \sum_{x_{t} \in \{A, C, G, T\}} p_{\theta}\left(\mathbf{x_{t} | x_{1 :(t-1)}}\right)=1  $$

Where *θ* represents the neural network parameter, the conditional probability *p*_*θ*_(*x*_*t*_|*x*_1:(*t*−1)_) can be obtained from the output of the neural network.

**Fig. 1:**
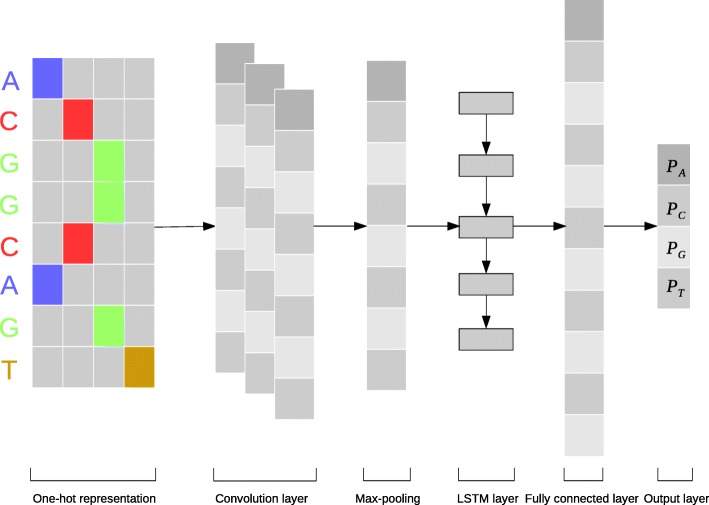
The architecture of the DeepDNA model. Firstly, the input genome sequence is transformed into one-hot 4-dimensions bit matrix; A convolution layer activated by a rectified linear units acts as a local feature extractor, its output is a matrix with column matrix of the convolution filter and the row matrix of the position in the input sequence; A max-pooling procedure is used to reduce the size of the output matrix and only preserve the main features; The subsequent Long Short-Term Memory network (LSTM) layer is considered as acting the role of capturing sequence long-term features; A flattened fully connected layer is to collect LSTM outputs; The last layer performs a sigmoid non-linear transformation to a vector that serves as probability predictions of the sequence base

The architecture of our neural network model DeepDNA is shown in Fig. 1, the framework mainly has six layers: the first layer is the single one-hot representation, which convert the genome sequence nucleotides {*A*,*C*,*G*,*T*} to vectors. The second convolution layer extracts the context short-term correlation in the genome. The third layer is the pooling layer to remove the noise. The fourth layer, LSTM, extracted the long-term correlation in the genome. The fully connected layer and the last layer are used for outputting next nucleotides {*A*,*C*,*G*,*T*} probabilities.

The following subsections describe how we apply CNN to extract genome sequences local features, LSTM to capture long-term dependencies over window features sequence and fed output layer to the arithmetic coder for getting the bit-stream respectively.

### Convolutional Neural Network (CNN)

One-dimensional convolution operation corresponds a series of filters sliding over the genome sequence identify the sequence characteristics at different positions in the genome, it takes the one-hot encoding of the genome as input, where one can be defined as:

4 $$\begin{array}{@{}rcl@{}}  \begin{array}{l} A \rightarrow [\!1, 0, 0, 0] \\ C \rightarrow [\!0, 1, 0, 0] \\ G \rightarrow [\!0, 0, 1, 0] \\ T \rightarrow [\!0, 0, 0, 1] \\ \end{array} \end{array} $$

Let $x \in \mathbb {R}^{T\times 4}$ represents the genome sequence with input length of T, and $x_{i} \in \mathbb {R}^{4}$ is the base vector representation of the *i*th position in the sequence. There are *m* filters in total, and the output of filters are $o \in \mathbb {R}^{(N-k+1)\times m}$, the convolution operation of each element *o*_*i*,*j*_ is defined as follows:

5 $$  o_{i,j} = \delta (w_{j} \odot [x_{i},x_{i+1}, \dots, x_{i+k-1}] + b_{j})  $$

where $w_{j} \in \mathbb {R}^{k\times 4}$ is the *j*th filter vector, and $b_{j} \in \mathbb {R}$ is a shared value for the bias for filter *w*_*j*_, symbol ⊙ is a convolution operation, define as follow:

6 $$  w_{j} \odot [x_{i},x_{i+1}, \dots, x_{i+k-1}] = \sum_{n=0}^{k-1}{w_{nj}x_{i+n}}  $$

Symbol *δ* is the neural nonlinear activation function, we select the ReLU [16] operation as the activation function, which outputs negative value to 0 and as defined below:

7 $$  ReLU(x) = \left\{ \begin{array}{rcl} x & \text{if} & x > 0 \\ 0 & \text{if} & x \leq 0 \end{array} \right.  $$

We use the max-pooling as the convolution layer after the output processing for the output vector feature extraction. Max- pooling operation selects the maximum value in an unit area as the representative feature of the sequence, which is used to extract the sequence higher scale features in the next layer.

To reduce over-fitting, a dropout layer is connected after the maximum pooling layer. The term “dropout” refers to the temporary deletion a apart of units in the neural network, deleting all the links associated with it. The probability of choosing which unit to be dropped is independent of the others, and with a fixed probability *p*.

### Long short-term memory networks (LSTM)

Recurrent neural network (RNNs) propagates historical message through a concatenation network structure, but has the problem of long gradient disappearance. Long Short-Term Memory Networks (LSTM) [17], as a special recurrent neural network, was proposed in 1997 to solve the problem that recurrent neural network (RNNs) cannot learn long-term dependent correlation. They are now widely used in various time series problems and have achieved a series of excellent results.

LSTM hierarchical transformation function is defined as follows:

8 $$\begin{array}{@{}rcl@{}}  f_{t} &=& \delta (W_{f} \cdot [\!x_{t}, h_{t-1}] + b_{f}) \\ i_{t} &=& \delta (W_{i} \cdot [\!x_{t}, h_{t-1}] + b_{i}) \\ o_{t} &=& \delta (W_{o} \cdot [\!x_{t}, h_{t-1}] + b_{o}) \\ c_{t} &=& f_{t} \odot c_{t-1} + i_{t} \odot tanh(W_{c} \cdot [x_{t}, h_{t-1}] + b_{c}) \\ h_{t} &=& o_{t} \odot tanh(c_{t})  \end{array} $$

Where $f_{t} \in \mathbb {R}^{h}$ is a forgetting gate to control which messages in the old memory unit will be discarded; $i_{t} \in \mathbb {R}^{h}$ is an inputting gate to control how much new messages will be recorded in the current memory unit; $o_{t} \in \mathbb {R}^{h}$ is an output gate to control output in the history unit. Through the joint control of the above three gates, the problem of long-term gradient disappearance is solved, and the dependence of long-term context can be excavated.

The terms *W*_*f*_, *W*_*i*_, *W*_*o*_, and *W*_*c*_ denote weight matrices for forgetting gate, inputting gate, outputting gate, and unit state connections. The terms *b*_*f*_, *b*_*i*_, *b*_*o*_, and *b*_*c*_ denote the bias vectors of the forgetting gate, inputting gate, outputting gate, and unit state connections. *x*_*t*_, *h*_*t*−1_ is the input sequence data for the current time and state output for previous time separately.

The symbol *δ* is function of logistic sigmoid, which limits output range to [ 0,1], defined as:

9 $$\begin{array}{@{}rcl@{}}  Sigmoid(x) = \frac{1}{1+e^{-x}} \end{array} $$

The function of *tanh* limits output range to [ −1,1], which is expressed as hyperbolic tangent function and defined by:

10 $$\begin{array}{@{}rcl@{}}  tanh(x) = \frac{e^{x} - e^{-x}}{e^{x} + e^{-x}} \end{array} $$

The symbol ⊙ represents the dot product operation of the element, as described in the formula 6.

### Arithmetic encoder

The arithmetic encoder [18] was proposed in 1976 by Rissanen and Pasco to solve the problem of infinite decimal precision, is a coding approach closest to information entropy when data distribution was fixed. Instead of encoding each character to an integer, the arithmetic encoder encrypts the sequence as a sufficiently precise numeric value in the interval (0,1), called sequence identifier or label. As the encoding progresses, the label interval becomes smaller and smaller, and the next interval range is fixed by the probability of encoding character. The decoding operation is similar to encode, given the character probabilities, the arithmetic decoder predict the probability to the corresponding character interval. As long as the decimal representation of the interval identifier or label is accurate enough, the decoder is able to recover the whole encoding sequence in lossless.

**Fig. 2:**
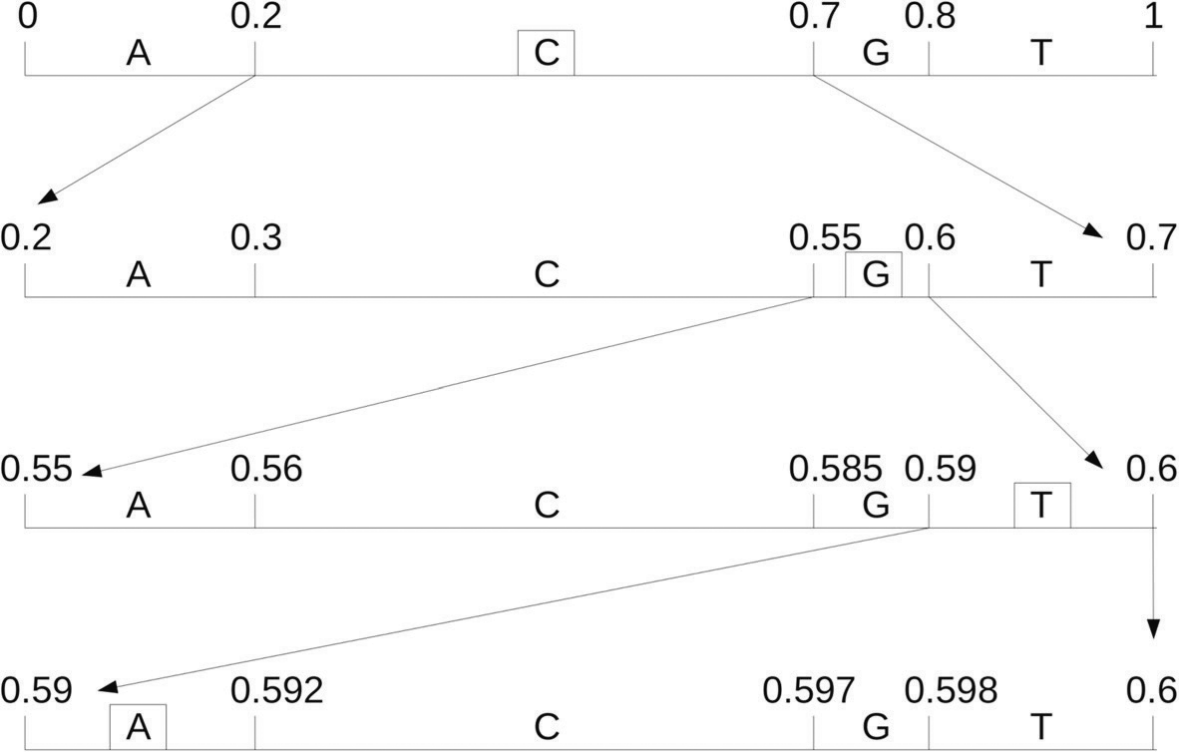
Arithmetic encoding process. It illustrates the sequence label determination process when encoding a sequence ’CGTA’, assume that the probability values of each base: *p*(*A*)=*p*(*T*)=0.2, *p*(*C*)=0.5, *p*(*G*)=0.1

Figure 2 demonstrates the identifier determination procedure of arithmetic encoder, for example, coding a sequence ‘CGTA’, we assume that the probability values of each base are: *p*(*A*)=*p*(*T*)=0.2, *p*(*C*)=0.5, *p*(*G*)=0.1. At beginning, the initial interval is (0,1), then the first base ‘C’ limited the interval to (0.2,0.7), base ‘G’ limited the interval to (0.55,0.6), and so on ⋯. The latter interval is a subset of the former interval, so the range will be more and more smaller, lastly, the identifier is limited the interval to [0.59, 0.592]. We can choose any point within this interval, like a middle value 0.591, its binary value stream is the arithmetic coded description of the raw sequence ‘CGTA’.

The decoding process is similar to encoding. First, base ‘C’ is decoded according to 0.591 within the interval (0.2,0.7), while the next decoding process 0.591 within the interval (0.55,0.7), the base ‘G’ is decoded, and so on ⋯, until decoded the last base ‘A’, and the whole sequence ‘CGTA’ is decoded.

Consider an input sequence *x*_*i*−1_,*x*_*i*−2_,…,*x*_*i*−*k*+1_ for the compression model, the output for the model is the predict the next base *x*_*i*_. The estimate probabilities *p*(*x*_*i*_|(*x*_*i*−1_,*x*_*i*−2_,…,*x*_*i*−*k*+1_),*h*_*i*−1_) is provided into arithmetic encoder to obtain the final output bit-streams, where *h*_*i*−1_ is the deep learning model former state. According to the theory of Shannon entropy [19], the number of output bits for the base *x*_*i*_ is determined by:

11 $$\begin{array}{@{}rcl@{}}  H = -{log}_{2}(p(x_{i}|(x_{i-1}, x_{i-2}, \dots, x_{i-k+1}), h_{i-1}) \end{array} $$

That is, the more accurate the probability of our model estimation, the higher the probability of corresponding coding base, the smaller the output bit-tream, and the better the compression effect we get.

### Model setting & training

In the deep learning model, we make comprehensive use of the local feature capture ability of CNN and the long-term feature extraction ability of LSTM. Our deep learning model implementation uses the Keras [20] library, which is an open resource for deep learning derived on the backend of Theano [21].

Comprehensive setting for the model structure are described as follows:

- Input layer (Input nucleotides: 64 ×4)
- Convolutional Neural Network (CNN) layer (Filters no.: 1024, window size: 24 ×4, stride: 1.)
- Max-Pooling layer (Window size: 3 ×4, stride: 1.)
- Dropout layer (Probability: 0.1)
- Long Short-Term Memory networks (LSTM) layer (LSTM units: 256)
- Dropout layer (Probability: 20%)
- Fully connected layer (Units: 1024)
- Sigmoid output layer (Units: 4)

In the deep learning model, all parameters are initialized according to random and uniform distribution *unif*(−0.05,0.05), and entire biases are initialized to 0. We use mini-batch training (default size: 64) to minimize the cross entropy loss function on the training data set. Validation losses were assessed at the end of each training epoch to monitor the convergence. We utilized about 10 epochs to complete the training, each of which took about ∼6 h.

The loss function of cross-entropy is defined as:

12 $$\begin{array}{@{}rcl@{}}  L(y, \widehat{y}) = -\frac{1}{n}\sum_{t=1}^{n}\sum_{i=1}^{4}{y_{i}}^{(t)} log(\widehat{y_{i}}^{(t)}) \end{array} $$

Where $\widehat {y_{i}}^{(t)}$ is the probability of prediction character at time *t* being nucleotide *i*, *y*_*i*_^(*t*)^ is the one-hot vector represent the real nucleotide at time *t*, and mini-batches sample size is *n*. We exploited the adaptive learning rate RMSprop [22] designed by Geoff Hinton as the learning rate of the model.

Both in the encoding and decoding process, it calculated the nucleotide probability based on the same deep learning network parameters, so they get the same prediction probability value, therefore, the original sequence can be lossless reconstructed by arithmetic coding.

## Results

### Dataset

In order to validate the effectiveness of our proposed model, 1,000 complete human mitochondrial genome sequences were used as experimental data, and the average sequence length of human mitochondrial genome sequence is 16,500bp. All data were download from the MITOMAP [23] database in the March 2019. We randomly selected 700 sequences being the training data set, 200 sequences being the verification data set, and 100 sequences being the test data set. In order to make the sequence consists of only 4 nucleotides (A, C, G, T), for simplicity, the fuzzy symbols was replaced with a fixed base “A” in the training process, and all lowercase nucleotides in the data set were converted to uppercase. In the compression process, we record the nucleotides not in the {*A*,*C*,*G*,*T*} and its position information, record whether the base is in lowercase or not, so that the original sequence can be reconstructed in lossless when decompressing.

### Results and model analysis

We experimented DeepDNA method for training set, verification set and test set respectively. The training set was utilized for learning model parameters, the verification set was utilized to determine network structure and model parameters, and the test set was utilized to verify the performance of the final selection of model parameters.

**Fig. 3:**
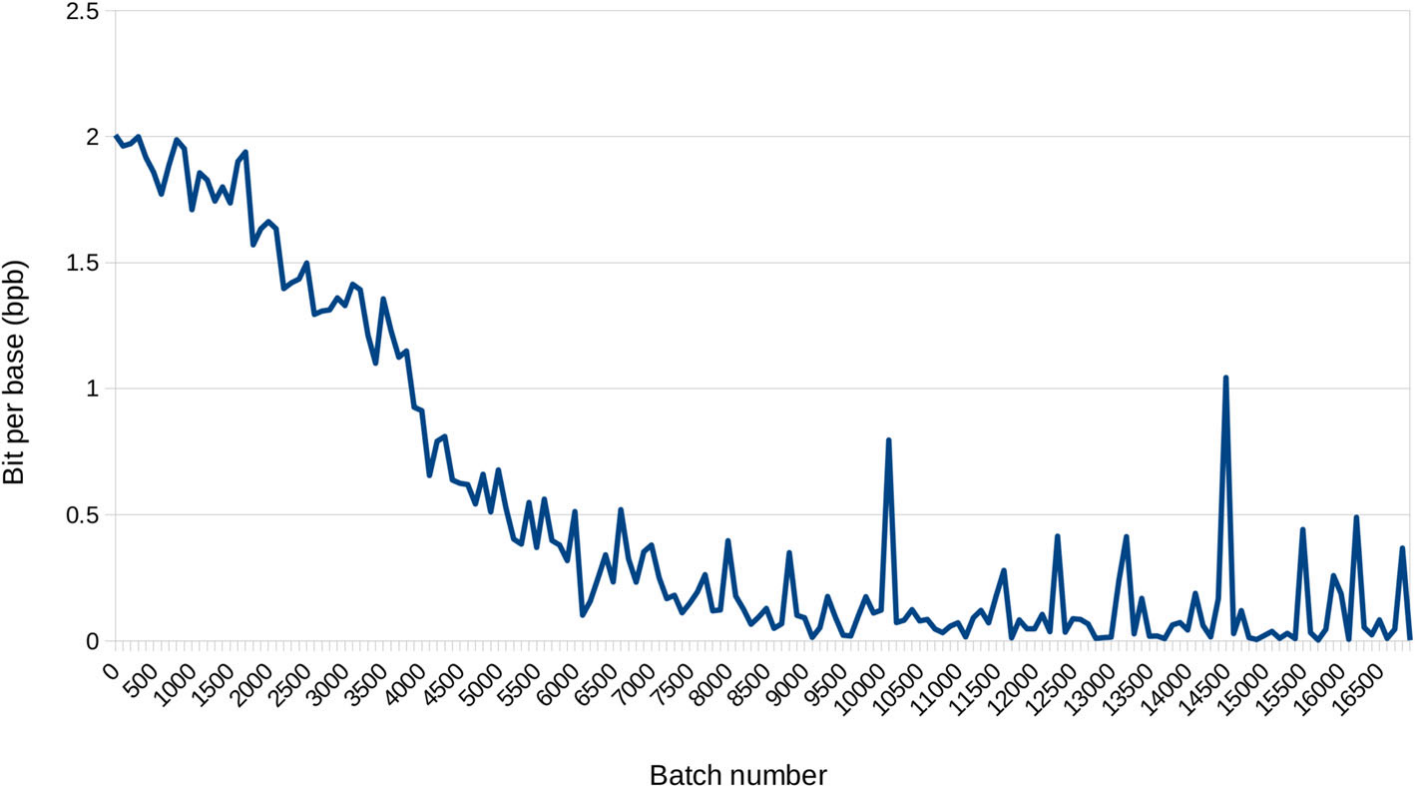
The training loss function values (bpb) as the number of training mini-batches for DeepDNA model. 700 human mitochondrial genome sequences were trained, and the input length of the base sequence was 64, and the output was the classification of the corresponding four nucleotides

As we can be seen from Fig. 3, with the increase of the number of training mini-batches, the loss function gradually decreases and ultimately tends to converge. There was a trivial fluctuation point at the final of training, owing to a few genomics structural variation sites, which will affected the prediction performance. However, these structural changes accounted for less than 1% of the total sequence, that is, a few differences in the data sites of human mitochondrial genome did not have much impact on the final compression results.

In order to verify the validity of our proposed DeepDNA model, we tested our method and the other four methods on the test set (100 human mitochondrial genome sequence data). Table 1 lists the compression results of the DeepDNA method, the Gzip [24] method, MFCompress [9] method, DMcompress [25] method, which we proposed earlier, the unit of compression results is in bits per base (bpb). The compression result of DeepDNA corresponds to the average of the lengths of all base prediction probability output codes in the genome sequence, namely:

13 $$\begin{array}{@{}rcl@{}}  DeepDNA (bpb) = -\frac{1}{T}\sum_{i=1}^{T} {log}_{2}(P(\widehat{y_{i}})) \end{array} $$

**Table 1:** Results for DeepDNA and the other methods compression for 100 human mitochondrial genomes

Dataset	Total size	Gzip	MFCompress	DMcompress	DeepDNA
	(nucleotides)	(bpb)	(bpb)	(bpb)	(bpb)
100 human					
Mitochondrial genomes	1,656,779	1.45	0.07	0.07	0.03

The measure of space occupied is evaluated in bits per base (bpb)

Where T is the sequence length of compression genome, and $P(\widehat {y_{i}})$ is the predicted probability value of the DeepDNA model output corresponding to the base *y*_*i*_ of the genome sequence at i-th position, which can be fed into arithmetic coding [26] directly and got the compression bit-streams file.

Table 1 shown that on the human mitochondrial genome test data set, the compression result of the normal text compression method Gzip is 1.45 bpb, and the DMcompress method proposed in our previous work and MFCompress method both are 0.07 bpb. The deep learning-based compression method DeepDNA proposed by us, has a compression result of 0.03 bpb. Our method DeepDNA has a better result to the other three methods in the human mitochondrial genome dataset.

Table 1 lists the results of compression of all 100 human mitochondrial genomes as a group data-set. To verify their efficiency of compressing on individual genome sequence, we randomly selected 5 human mitochondrial genomes. The data is independently compressed and compared the results. The Table 2 lists the compression results of the 5 human mitochondrial genome sequences. It can be seen from the table that our proposed DeepDNA method achieves the best compression results on all five independent genomes. Comparing with the current normal text compression method Gzip, the finite context model based compression method MFCompress, and our earlier information entropy-based compression method DMcompress, the result of compression of DeepDNA is less than 0.05 bpb.

**Table 2:** Detailed results for DeepDNA and the other methods on randomly selected five sequences from 100 human mitochondrial genome sequences

Genome ID	Gzip (bpb)	MFCompress (bpb)	DMcompress (bpb)	DeepDNA (bpb)
KF162105.1	2.63	2.09	2.07	0.01
MF058266.1	2.64	2.09	2.07	0.05
KC911416.1	2.64	2.09	2.06	0.01
AY339411.1	2.63	2.09	2.07	0.01
JQ702777.1	2.64	2.08	2.06	0.04

The measure of space occupied is evaluated in bits per base (bpb)

## Discussion

The Tables 1 and 2 show that our proposed method can not only achieve compression effect on the multi-genomes, but also achieve good compression effect on individual genome data. The other three compression methods have better compression on the multi-genomes than on the individual genome, because the data redundancy on the multi-genomes is higher than the individual genome. The neural network model we proposed for lossless genome compression is not affected by this limitation.

The neural network parameters, as part of the compression model, obtained through training and learning directly participate in the compression decompression process. Thus it avoided the process of continuing to update parameters during compression/decompression, saving a lot of time. Because of the consistency between genomes, we can train the compression model in advance, and then used directly for compression.

## Conclusions

We designed a novel, machine learning method, DeepDNA, which integrates the convolutional neural network (CNN) and the long short-term memory network (LSTM) for compressing the genome sequences. Experiment on 1,000 complete human mitochondrial genome sequences have shown that our method can learn local features of sequences through convolution layer, and can learn advanced representations of long-term dependence of sequences through long short-term memory network (LSTM). We evaluated the performance of deep learning model on 100 human mitochondrial genome sequences compression task and obtained an acceptable result.

Our model indicated the feasibility of compressing genome sequences via CNN and LSTM network models. This work will help to better explore the patterns and rules in genome sequences, assist in decoding the functional characteristics of sequences, and to help resolve the link between genes and disease. Because better sequence prediction model will be achieved better compression effect, and a better sequence prediction model can help solve all above problems.

In addition, with the exploration of genome sequence characteristics in future biological analysis, the compression method can obtain more redundant information, and get a better improved compression performance. In the next work, we can explore methods of lossless compression of the entire human genome, making full use of as much background information as possible, for instance, mutations, tandem repeats, motifs, etc., to train the machine learning model for compression genome sequences.

## Data Availability

The dataset generated and analysed during the current study are available in the Mitomap repository, https://www.mitomap.org/MITOMAP
